# Teaching and Practicing Cognitive-Behavioral and Mindfulness Skills in a Web-Based Platform among Older Adults through the COVID-19 Pandemic: A Pilot Randomized Controlled Trial

**DOI:** 10.3390/ijerph182010563

**Published:** 2021-10-09

**Authors:** Stav Shapira, Ella Cohn-Schwartz, Daphna Yeshua-Katz, Limor Aharonson-Daniel, Avram Mark Clarfield, Orly Sarid

**Affiliations:** 1 School of Public Health, Faculty of Health Sciences, Ben-Gurion University of the Negev, Beer Sheva 8410501, Israel; limorad@bgu.ac.il; 2 PREPARED Center for Emergency Response Research, Ben-Gurion University of the Negev, Beer Sheva 8410501, Israel; 3 The Department of Public Health, Faculty of Health Sciences, Ben-Gurion University of the Negev, Beer Sheva 8410501, Israel; ellasch@bgu.ac.il; 4 Department of Communication Studies, Faculty of Humanities and Social Sciences, Ben-Gurion University of the Negev, Beer Sheva 8410501, Israel; yeshuad@bgu.ac.il; 5 Medical School for International Health, Faculty of Health Sciences, Ben-Gurion University of the Negev, Beer Sheva 8410501, Israel; markclar@bgu.ac.il; 6 The Department of Geriatrics, McGill University, Montreal, QC H3T 1E2, Canada; 7 The Spitzer Department of Social Work, Faculty of Humanities and Social Sciences, Ben-Gurion University of the Negev, Beer Sheva 8410501, Israel; orlysa@bgu.ac.il

**Keywords:** COVID-19, depression, loneliness, social support, web-based group intervention, randomized controlled trial

## Abstract

The outbreak of the COVID-19 pandemic has led to an acceleration in the development of web-based interventions to alleviate related mental health impacts. The current study explored the effects of a short-term digital group intervention aimed at providing cognitive behavioral and mindfulness tools and skills to reduce loneliness and depression and to increase social support among older adults in Israel. This pilot randomized controlled trial included community-dwelling older adults (*n* = 82; aged between 65–90 years; 80% female) who were randomized either to an intervention group (*n* = 64) or a wait-list control group (*n* = 18). The intervention included seven online sessions, over 3.5 weeks. Depression, loneliness, and social support measures were administered at baseline, immediately post-intervention, and at 1-month follow-up. Repeated measures ANOVA revealed statistically and clinically significant reductions in depression in the intervention group, with results maintained at one-month follow-up. Loneliness levels also significantly decreased post-intervention; however, this benefit was not maintained at one-month follow-up. Social support slightly increased both post-intervention and 1-month follow-up—but these changes were not statistically significant. There were no overall changes for the wait-list control group. Our intervention provided promising evidence regarding the effectiveness of an online group intervention to alleviate mental health effects and to promote the coping of older adults during the COVID-19 pandemic. This relatively simple model can be effectively utilized by communities globally to help connect lonely and isolated older inhabitants, both during the pandemic and in more routine times.

## 1. Introduction

The coronavirus disease 2019 (COVID-19) poses clear increased risks for the older population, who face heightened dangers of illness and death [1]. Alongside the health perils, older adults also face increased social isolation as they are asked to maintain physical distancing and limit their social interactions to protect their health as well as to protect the health care system from being overwhelmed [2]. However, social contact is a fundamental human need and is considered an important factor for maintaining physical, mental, and cognitive health [3,4]. Recent evidence has indicated the importance of using strategies that provide regular social contact with family or friends in order to maintain a good perception of subjective health among older adults [5]. Thus, limiting social contacts during the pandemic can have far-reaching negative consequences for the mental and physical health of older adults [6,7]. One of the main concerns related to protective measures such as social distancing during the COVID-19 outbreak, especially among older individuals, has been an increase in loneliness. Indeed, research indicates an increased level of loneliness among older adults during the pandemic, especially those adhering to the physical distancing measures [8,9]. Older individuals can suffer from increased distress during the pandemic, not only from social isolation but also from the usual threats to health which might not be attended to and from the overall uncertainty brought about by these stressful times. Some older adults have indeed shown elevated levels of negative mental reactions (e.g., depression and anxiety) following the COVID-19 pandemic [10]. 

The need for relevant solutions was stressed by the World Health Organization (WHO), which called for communities to ensure that older adults are not being overly isolated or placed in a position of increased vulnerability during the outbreak. This guidance includes helping older persons stay connected with others through various means [11]. As such, societies have an obligation to maintain the connectedness of older adults, especially as they are asked and advised to stay physically isolated. Investigation is required to produce effective coping measures and to establish interventions which will decrease the risks of social isolation and mental distress of older adults in light of this global crisis as well as for cases of future outbreaks of communicable diseases [12,13].

Indeed, this pandemic has accelerated the development of interventions using information and communication technologies (ICT) to help connect lonely and isolated older adults during COVID-19 and beyond [14]. ICT interventions can help adults adhere to necessary restrictions and the need to physically isolate, thereby protecting their health while still allowing for the creation and maintenance of social connections and the exchange of social support [15,16,17]. Moreover, the utility of such interventions goes beyond the time of a pandemic, as many older adults may live in remote areas, be homebound, or not have the financial and transportation possibilities to attend in-person groups in the community [18,19].

Digital interventions also allow for teaching therapeutic skills and techniques that can be practiced by older adults to alleviate distress and to promote effective coping [20]. Existing digital interventions that address mental health issues among older adults often use mindfulness or Cognitive Behavioral Therapy (CBT) approaches. For example, a short-term (one session) cognitive-behavioral and art (CB-ART) intervention was reported to reduce subjective distress among participants during the pandemic’s first lockdown in Israel [21]. Online interventions are usually used by single individuals in either a guided or self-guided manner [20,22,23]. The effectiveness of such interventions could be enhanced in group settings that can promote the adoption of such skills and techniques by offering peer advice, role modeling, and accountability. They are also efficient, given that one instructor is shared by several participants [24]. The pandemic, therefore, provides a unique opportunity to add and evaluate elements of group interventions, which are regularly implemented in face-to-face settings to online mental health programs for older adults.

Interventions with older adults in the times of this pandemic should also strive to decrease loneliness and to improve social support. Prior research indicated several guiding principles in the design of such interventions. Loneliness can be differentiated into chronic and situational loneliness, both of which have negative consequences [25]. The pandemic increases situational loneliness, and interventions that foster social connections and social support can be more effective compared with interventions that teach social skills, which are more relevant for chronic loneliness [26]. A meta-analysis of programs meant to reduce loneliness indicated the usefulness of group interventions in addition to those that include active member participation or social activities, compared with one-on-one interventions [27].

ICT can be used to address the risk of loneliness in this context. The convoy model of social relations emphasizes the importance of social connections in old age and specifies the various benefits of technology to improve the availability of social support [28]. The use of technologies in interventions was deemed useful in reducing loneliness among older people by several systemic reviews [29,30,31]. However, the technologies reviewed mostly focused on companion robots or internet training to promote contact with existing family and friends, for example, via social networking sites. Such interventions are lacking the advantages of guided group settings, in which members provide companionship and support. At the onset of COVID-19, the transfer of group interventions to an online setting still remains a challenge.

Despite the growing need for online interventions during the pandemic, there are only few relevant studies that documented the adaptation and development of ICT interventions for older adults during COVID-19. These include the ICT adaptation of a social support group intervention [14] and of a group cognitive stimulation therapy for people with dementia [32] in addition to a protocol for a volunteer-based telehealth intervention program [33]. However, while forming a basis for program development, the efficacy of these programs was not assessed. An empirical investigation was performed regarding a telephone contact intervention, which yielded promising results, but the protocol itself was limited to a one-on-one single-time contact and its effects were measured only via volunteer reports [34].

A more rigorous assessment of an ICT intervention came from a study on the effects of an online group intervention for older adults during COVID-19, which is also at the focus of the current study. That previous work yielded positive outcomes in terms of depressive symptoms and loneliness [35]. However, it was limited by only measuring participants immediately following the program’s termination, while lacking a more comprehensive account of longer lasting effects. Additionally, it only measured depressive symptoms and loneliness, while not assessing social support, although it could also be impacted by such a program. The current study expands these previous results and explores the relatively long-term effects of the intervention on a wider set of outcomes.

The current study aims to examine the long-term effects of a group intervention via a pilot RCT based on a videoconferencing app (Zoom) among community-dwelling older adults. It was developed to teach and practice CBT and mindfulness techniques and skills to enhance coping abilities and to reduce distress during the pandemic as well as to foster group discussions and contacts to alleviate loneliness and to improve social support. It was implemented during the initial months of the COVID-19 pandemic, a time in which Israel underwent its first lockdown and subsequently began to ease restrictions. The study assessed participants before the intervention, immediately following its termination and one month afterwards, compared with a control group that did not undergo the intervention. We were guided by three hypotheses:

1. Compared with the control group, participants in the intervention group report decreased depressive symptoms immediately and one month following the intervention.
2. Participants in the intervention group report decreased loneliness immediately and one month following the intervention.
3. Participants in the intervention group report increased social support immediately and one month following the intervention.

## 2. Materials and Methods

This pilot-RCT tested an internet-based group intervention to alleviate loneliness and depressive symptoms among older adults compared with a wait-list control group. The intervention consisted of seven sessions over 3.5 weeks. Participants comprised a convenience sample of 82 community-dwelling individuals in Israel. They were aged 65–90 (M = 72, SD = 5.6) and had to be fluent in Hebrew. Additional inclusion criteria were having an active internet connection, possessing at least one device enabling online communication, and having a minimal ability to operate this device (i.e., turning it on and off). Participants were recruited over 4 months between March and June 2020 through an online invitation that was circulated in WhatsApp groups established by a local non-governmental organization that focuses on enhancing digital literacy among seniors. Furthermore, we recruited participants with the assistance of relevant local welfare departments responsible for the social care of older adults in several municipalities in Israel. All participants provided written informed consent, and the study was authorized by the Ben-Gurion of the Negev IRB.

### 2.1. Procedure

**Figure 1:**
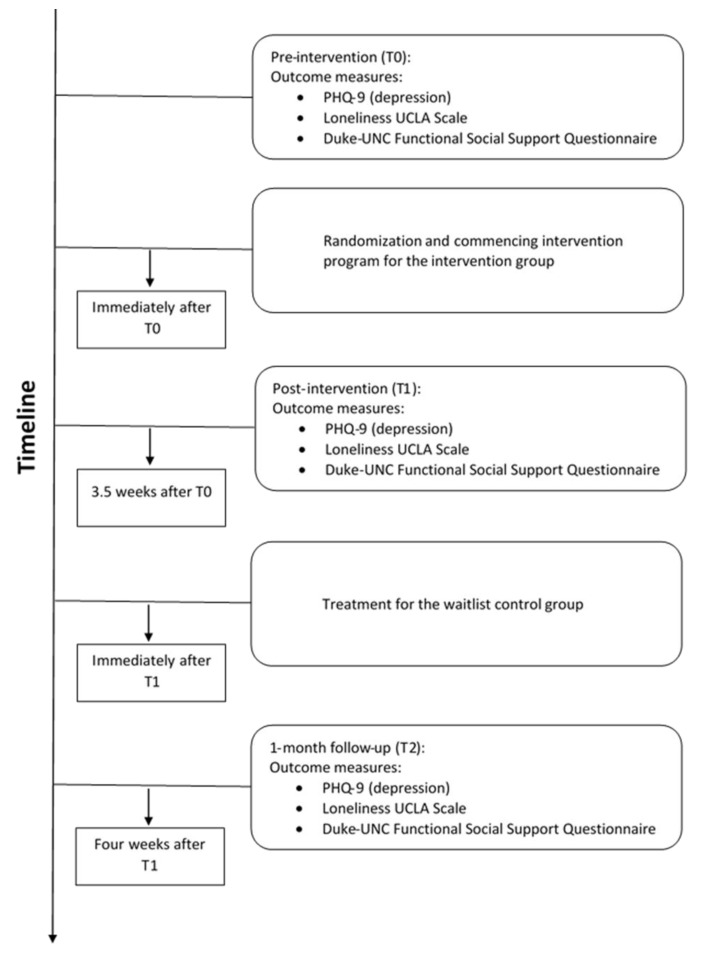
Timeline for the intervention and measurement points.

Participants in the intervention arm (*n* = 64) were assessed at baseline (T0), post-intervention (week 4—T1), and 1-month following the end of the intervention (week 8—T2) (see Figure 1). Participants in the wait-list control group (*n* = 18) were assessed twice before entering the intervention group and at T1 and T2. Participation in the program was voluntary, and the participants were not compensated for it. Following approval by the Institutional Review Board, treatment and control groups commenced in April 2020 and were completed by July 2020. 

### 2.2. Group Allocation

A research coordinator blind to the hypotheses independently performed the allocation process, using a table of random numbers with no further constraints and without participant contact. The participants were randomized via a 4:1 ratio into either intervention or wait-list control group, respectively. We used this allocation instead of an even ratio, which is the more classic RCT ratio due to ethical considerations; we wanted to provide mental support as quickly as possible to as many people that were, at the time (during the initial months of the COVID-19 pandemic), isolated at their home for an unknown period due to the pandemic. Participants were told that they would be participating in a program that aims to provide them with tools and skills to better cope with the stressful situation caused by the pandemic and to provide them with a safe virtual space to share hardships in a supportive atmosphere. Those who were allocated to the wait-list control group were instructed that they would be assigned to a group within the next 4 weeks.

### 2.3. Intervention

Detailed information regarding the intervention protocol and content was previously reported elsewhere [35,36]. In brief, online sessions via the Zoom videoconferencing platform were delivered to groups of 5–7 people during seven sessions over 3.5 weeks each lasting between 1–1.5 h. That is, in each of the first three weeks, the participants attended two sessions, and during the fourth week, they met once for the concluding session. The instruction was delivered by four moderators, all of whom were clinical social workers trained to guide the program by a senior clinical social worker from the research team with experience in cognitive behavioral interventions. Clinical supervision was conducted weekly via Zoom during the intervention period. In parallel to the group sessions, each moderator established a WhatsApp group for the participants in his/her group in order to allow for ongoing communication between the participants themselves and/or with the moderators during and between sessions. These WhatsApp group were also used as a platform for learning, via sending supplementary materials (text, video, and audio files) for practicing the techniques taught in the sessions.

The two main components of the intervention were (a) guided group discussions, and (b) learning and practicing CBT techniques and skills (e.g., breathing, guided imagery of a ‘safe place’, constructing positive self-talk, mindfulness meditations, and more) aimed at identifying non-adaptive cognitive schemes and developing capacities to promote better coping. The collection of therapeutic techniques in our intervention protocol is based on recent evidence indicating that combined mindfulness-CBT programs were found effective in assisting people to cope with a variety of health conditions such as anxiety [37], insomnia [38], and Crohn’s disease [39].

#### Wait-List Control Group

Participants who were allocated to the wait-list control group did not receive any treatment. A research assistant contacted each participant via telephone in the second week of their waiting in order to provide details about their future allocation into a treatment group.

### 2.4. Outcome Measures

#### 2.4.1. Depression

Depression and severity of relevant symptoms were assessed using a nine-item depression severity measure. This measure is part of the Patient Health Questionnaire (PHQ-9) and is used as a diagnostic instrument for common mental disorders [40]. The PHQ-9 scores each of The Diagnostic and Statistical Manual of Mental Disorders, Fifth Edition (DSM-V), criteria as 0 (*not at all*) to 3 (*nearly every day*). The responses were summed, resulting in a score ranging between a minimum of 0 and a maximum of 27. Scores of 5, 10, 15, and 20 represent mild, moderate, moderately severe, and severe depression, respectively. The scale includes such items as “Over the last 2 weeks, how often have you felt down, depressed or hopeless?”. The last item of the PHQ-9 targets suicidal inclination and is utilized as a screening measure for suicidality in primary care. The PHQ-9 has previously been tested among the Israeli population in Hebrew with good reliability (Cronbach’s alpha ranged between 0.88 to 0.93) [41].

#### 2.4.2. Loneliness

We used the Short Scale for Measuring Loneliness [42], which comprises three items examining perceptions related to lack of companionship (“How often do you feel lack of companionship?”), social exclusion (“How often do you feel left out?”), and social isolation (“How often do you feel isolated from others?”). The response categories are coded as 1 (*hardly ever*), 2 (*some of the time*), and 3 (*often*). The responses are summed, resulting in a total score ranging 3 to 9, with higher scores indicating greater loneliness. This scale has been previously used among the Israeli population in Hebrew and showed good reliability (Cronbach’s alpha was 0.87) [43]. 

#### 2.4.3. Social Support

We assessed this element using the Duke-UNC Functional Social Support Questionnaire [44], a validated tool for measuring social support that has been validated among various populations, among them older adults [45]. The tool contains eight items, rated on a five-point Likert scale, that address both emotional support and functional support. Examples of questions are “I have people who care what happens to me, I get love and affection”, etc. An average score is calculated, resulting in a total score ranging between 1 and 5: The higher the average score, the greater the perceived social support. This measure has previously demonstrated adequate levels of reliability (Cronbach’s alpha of approximately 0.80–0.85) [46].

### 2.5. Sample Size Calculation

The sample size calculation, using 2 (group) × 3 (time) mixed-model repeated measure ANOVA, was based on a review of similar internet-based interventions to alleviate loneliness and depression [47]. Assuming an effect size of f = 0.25 and α-probability of 0.05 yielded a minimum sample of 44 participants. Considering dropout and retention [48], 124 participants were recruited. The sample size calculations were performed with G*Power 3.1 software.

### 2.6. Statistical Analyses

A series of 2 (group) X 2 (time) repeated-measures mixed ANOVAs examined the change in depression, loneliness, and social support over the measurement points among participants in the intervention program and the wait-list control. Effect sizes are reported as partial *η*2 coefficients. To further interpret any time by-group interactions and to examine whether the gains from participation maintained at 1-month follow-up, a series of paired t tests examined potential differences between baseline and post intervention (T0 and T1), between post intervention and 1-month follow-up (T1 and T2), and between baseline and 1-month follow-up (T0 and T2). Participants who did not fill any questionnaire or were never allocated to the intervention group were excluded from the analysis. All statistical analyses were conducted using SPSS (version 26, SPSS Inc., Chicago, IL, USA). 

### 2.7. Clinically Meaningful Improvement

Clinically meaningful improvement was defined as a reduction between T0 and T2 of at least five points of the baseline PHQ-9 score [49] and related only for the depression measure (PHQ-9). Chi square analyses were used to test between-group differences in the proportion of participants showing clinically significant improvement.

## 3. Results

### 3.1. Recruitment and Attrition

Following the circulation of the invitation to participate in the study, 124 applicants indicated an initial interest. Of these, 86 met our inclusion criteria and were randomized (31% non-eligible due to: age <65 or non-response), and 82 ultimately provided data for the study—68 in the intervention arm, 18 in the wait-list control (95% of eligible). Thirteen participants withdrew from the intervention group, leaving 55 participants who were originally allocated to the intervention are and finished the program (68 − 13 = 55). Nine participants withdrew following the end of the waiting period (17% attrition in the control group), leaving nine participant who commenced the intervention. That is, the final group that provided pre- and post-intervention data included 64 (55 + 9) participants. Chi square and *t*-test analyses showed no significant group differences between those participants who completed the intervention and those who dropped out early—either in demographics or in baseline scores of study measures (*p* > 0.05).

### 3.2. Baseline Characteristics

Table 1 provides a summary of the participants’ demographic characteristics and baseline scores of study measures, stratified by study group. No significant differences were found between the two groups. The participants were mostly women, and they were aged average 72 years. Over a third lived alone, and a majority (72.6%) has tertiary education.

Table 2 details the scores of study measures at all three time points for both intervention and wait-list control group.

### 3.3. Changes in Depressive Symptoms

**Figure 2:**
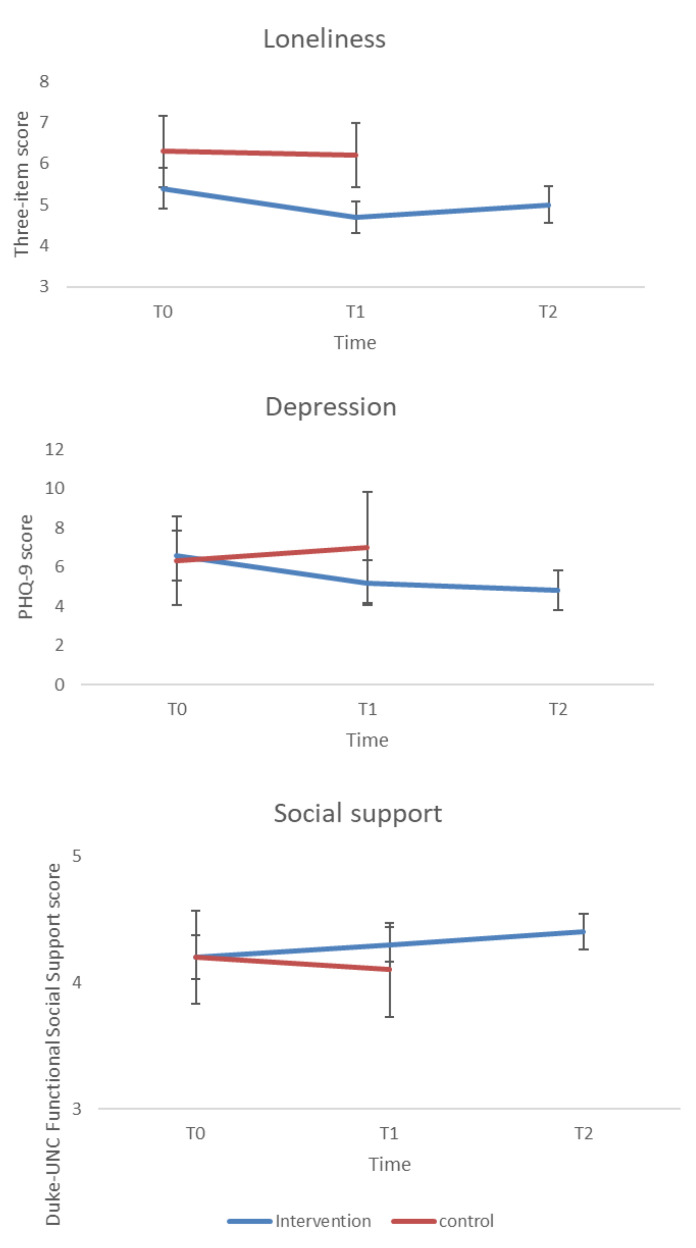
Outcomes measures of loneliness, depression (PHQ), and social support scores from baseline measurement (T0), end of program (T1) and 1-month follow-up (T2) in the intervention group and the wait-list control group. The graphic presents mean values and 95% confidence interval.

The ANOVA testing for changes in depressive symptoms demonstrated a marginally significant main effect of time-by-group interaction (F(1, 79) = 3.82, *p* = 0.05, *η*2 = 0.05), indicating a marginally significant difference between the groups post-intervention. The main effect of time (F(1, 79) = 0.35, *p* = 0.55, *η*2 = 0.00) did not reach statistical significance. Plot inspection confirmed by *t*-tests (Table 3 and Figure 2) showed that the intervention significantly reduced depression scores between T0 and T1, with these relatively lower scores maintained at T2. No changes in depression scores were observed in the wait-list control group between T0 and T1.

### 3.4. Clinically Significant Changes in Depressive Symptoms

Analyses demonstrated group difference in the proportion of participants showing clinically meaningful improvement between T0 and T2. In the intervention group, 10 (16%) participants recorded a clinically meaningful decrease in PHQ-9 depression scores post-intervention (T1) and this improvement was maintained among 7 (10%) participants at 1-month follow-up (T2), compared with only 1 (5%) participant in the wait-list control group.

### 3.5. Changes in Loneliness

The ANOVA showed a significant main effect of time-by-group interaction (*F*(1, 78) = 5.59, *p* = 0.02, *η*2 = 0.07). The significant interaction indicated that the groups differed in loneliness scores post-intervention. The main effect of time did not reach statistical significance (*F*(1, 78) = 0.57, *p* = 0.45, *η*2 = 0.001). Plot inspection, confirmed with t-tests (Table 3, Figure 2) showed that loneliness decreased in the intervention group between T0 and T1; however, this gain was not maintained at T2 (*p* > 0.05), although the score in this measurement was still lower compared with T0 (M = 5.4, SD = 2 vs. M = 5, SD = 1.8). No changes in loneliness were found in the wait-list control group.

### 3.6. Changes in Social Support

The results of the ANOVA tests did not show any significant effects for time (*p* = 0.44), group (*p* = 0.43), or for the time-by-group interaction (*p* = 0.10). Plot inspection, confirmed with t-tests, showed a slight increase in perception of social support (both between T0 and T1 from M = 4.2, SD = 0.7 to M = 4.3, SD = 0.6, and between T1 and T2 from M = 4.3, SD = 0.6 to M = 4.4, SD = 0.6); however, these changes were not statistically significant (Table 3 and Figure 2).

## 4. Discussion

The current study used an RCT methodology to examine the effectiveness of an online group intervention during the initial months of the COVID-19 pandemic and the resulting lockdown in a convenience sample in Israel. This global crisis with resultant physical isolation imposed in particular on older persons emphasized the need for online group interventions that can be easily administered by communities. Such interventions should address the mental health and social needs of older adults while maintaining social distancing guidelines [11]. The intervention model studied here combined several coping-related cognitive-behavioral and mindfulness strategies and the fostering of social connectedness in an online group setting. The results indicated that the group outcomes persisted for at least one month in terms of depressive symptoms, in accordance with the first study hypothesis. Loneliness scores decreased immediately following the intervention; however, after one month, they were no longer significantly lower compared with T0, in partial agreement with the second study hypothesis. The third hypothesis was not confirmed as social support did not significantly change following the intervention.

The different effects on depressive symptoms, loneliness, and social support may hint at the mechanisms underlying the intervention. Depressive symptoms remained low immediately and one month after the intervention, when comparing the intervention to the control groups. This result can attest to the effectiveness of the coping CBT techniques taught during the meetings. An advantage of such techniques is that they can be practiced in private and do not necessitate a group setting [50]. The current interventions further promoted such an independent implementation by sending supplementary materials (text, video, and audio files) to participants so they may utilize these techniques at home. While it is possible that depressive symptoms declined at least in part due to the easing of restrictions in Israel, the finding that the control group did not improve at T1 as well as the lack of a continued decline in loneliness all suggest that this trend is related primarily to the intervention. In addition, the clinical improvement documented among some of the participants suggests that our interventional model can be integrated into routine treatment programs of people with clinical depression; however, further research is required to determine if this is the case.

The somewhat different trend for loneliness and social support may attest to the different effects of the group aspect of the intervention. The intervention was set in a group context, which met regularly and which actively promoted social connections through guided group discussions among its participants. Thus, in addition to teaching relaxation and coping techniques, is also set out to address the social isolation brought about by the COVID-19 restrictions. The decrease in loneliness from T0 to T1, seen only in the intervention group, suggests that such regular contacts were indeed helpful in alleviating at least some of the loneliness that resulted from the pandemic, strengthening the notion that digital group interventions are effective in relation to this goal [51]. This intercession was especially important at the height of the restrictions, when social clubs were closed and face-to-face meetings were not allowed. However, the lack of a continued decrease in loneliness after one month may underscore that the group contacts themselves led to the decline in loneliness, and once terminated, participants returned to their “regular” life with less frequent interactions. This may indicate that social interventions are helpful in alleviating loneliness but only insofar as they are sustained over time. Thus, future programs should strive to promote continuous long-term contacts between participants even after the end of the intervention.

Social support was not significantly changed, although there was a nonsignificant trend towards increased support. Perhaps a future study with a larger sample size will show a significant trend. Additionally, other indicators such as social integration may show a more substantial increase following such an intervention [52].

The pandemic made ICT more present in the lives of older adults and accelerated the existing trend of a rise in the adoption of digital technologies among older adults [53]. However, some older adults may still fear that their technological skills present a barrier to their participation in ICT interventions [54]. Such concerns were partly reflected in our study sample, as not all of the adults enrolled in the intervention were technologically proficient. Research assistants were available to provide them with technical support and guidance on operating the Zoom app, and this helped those older adults who faced technological difficulties to successfully take part in the group meetings. Thus, our findings also suggest the benefits of an online intervention even among adults who may initially find technology intimidating.

The present study has both theoretical and practical implications. From a theoretical perspective, the current findings contribute to the understanding of acceptance of web-based interventions by older adults and to the study of how therapeutic interactions mediated by technology can shape the lives of older adults during public health emergencies. From a practical point of view, our pilot study identifies critical considerations that need to be taken into account when developing web-based mental health interventions: for example, the appropriate duration of such interventions and the issue of adequate technical support that must be ensured in order to include participants with low digital literacy. In order to advance the study in the developing discipline of web-based interventions, future multiple-armed RCTs should investigate the effects of alternative treatments, e.g., guided interventions vs. self-help interventions, or should test the effectiveness of different types of therapeutic tools and techniques and of various treatment durations (i.e., number and frequency of sessions) on the same outcome measures.

Several limitations of the study should be noted. One limitation concerns the control group not being followed at T2. However, the acute pandemic situation necessitated the quick allocation of all the interested older adults into the group intervention. Thus, it was deemed ethical to provide these adults with a much-needed intervention after only 4 weeks “untreated” rather than wait for one more month, which they could have spent in isolation and mental health decline. An additional limitation is the relatively small sample size, as this was a pilot study. While our findings are encouraging, their efficacy should be further validated in a larger RCT [51]. Another limitation is that the follow-up measurement did not include an assessment of participants’ extent of usage in the acquired techniques and skill during the period between the end of the program and the final measurement; this may potentially cause a certain bias in the results of T2. Finally, our intervention was multi-faceted, and some aspects may have influenced others, most probably positively but, at least in theory, negatively as well. Only further larger studies allowing for subgroup analyses will enable us to determine the relations if any among different program elements.

## 5. Conclusions

This pilot-RCT offers promising evidence regarding the effectiveness of an online group intervention to promote the coping of older adults during the COVID-19 pandemic, while maintaining their health and safety. Such an intervention can be used by communities globally to provide solutions to their older inhabitants, both during the pandemic and in routine times. The structured format of the intervention makes it relatively simple and appealing for practitioners and community organizations to implement [32]. Intervention programs should pay particular attention to older adults who live alone who may suffer more from social isolation and find it especially difficult to maintain social contacts during the pandemic [55]. Such persons, in addition to those who live in remote areas and are homebound [18,19], would greatly benefit from the further development of online group interventions that can reach a wider array of older adults and would help to improve their mental health.

## Figures and Tables

**Table 1:** Baseline characteristics of the sample.

	Intervention Group (*n* = 64)	Wait-List Control Group (*n* = 18)	*p* Value	Range
Sociodemographic characteristics				
Gender (female), *n*(%)	52 (81%)	14 (78%)	0.74	---
Age (years), M(SD)	72.1 (5.3)	71.7 (6.8)	0.79	65–90
Living alone, *n*(%)	24 (37.5%)	6 (35%)	0.86	---
Tertiary education, *n*(%)	48 (76%)	10 (59%)	0.15	---
Study measures, M(SD)				
Loneliness	5.4 (2)	6.3 (1.9)	0.21	3–9
Depression (PHQ-9)	6.6 (5.2)	6.3 (4.9)	0.85	007–27
Social support	4.2 (0.7)	4.2 (0.8)	0.95	1–5

**Table 2:** Primary outcomes at T0, T1, and T2.

	To	T1	T2
Measure	M (SD)	M (SD)	M (SD)
Loneliness			
Intervention	5.4 (2.0)	4.7 (1.6)	5.0 (1.8)
Wait-list	6.3 (1.9)	6.2 (1.7)	NA
Depressive symptoms			
Intervention	6.6 (5.2)	5.2 (4.7)	4.8 (4.1)
Wait-list	6.3 (4.9)	7.0 (6.1)	NA
Social support			
Intervention	4.2 (0.7)	4.3 (0.6)	4.4 (0.6)
Wait-list	4.2 (0.8)	4.1 (0.8)	NA
**Clinical**			
Depressed, *n*(%)			
PHQ ≥ 12 *			
Intervention	15 (23)	7 (11)	6 (9)
Wait-list	2 (11)	5 (27)	NA

* significant difference between groups at baseline, *p* < 0.05.

**Table 3:** The results of t tests between T0 and T1, and T1 and T2, and the overall differences between T0 and T2 for intervention and wait-list control groups separately.

Measure	T0 and T1	Difference, M(SE)	T1 and T2	Difference, M(SE)	T0 and T2	Difference, M(SE)
Loneliness						
Intervention	t(63) = 2.15 *	−0.7 (0.28)	t(63) = −0.88	0.3 (0.26)	t(63) = 1.5	−0.4 (0.24)
Wait-list	t(17) = 0.34	−0.1 (0.32)	NA	NA	NA	NA
Depressive symptoms						
Intervention	t(63) = 2.57 *	−1.4 (0.48)	t(63) = 1.15	−0.4 (0.38)	t(63) = 3.3 *	−1.8 (0.53)
Wait-list	t(17) = −0.72	0.7 (1)	NA	NA	NA	NA
Social support						
Intervention	t(63) = −1.03	0.1 (0.06)	t(63) = −0.39	0.1 (0.06)	t(63) = −1.31	0.2 (0.07)
Wait-list	t(17) = 1.08	−0.1 (0.16)	NA	NA	NA	NA

* Significant difference between groups at baseline, *p* < 0.05.

## Data Availability

The data presented in this study are available from the corresponding author upon request. The data are not publicly available due to privacy considerations.

## References

[1] Wu C., Chen X., Cai Y., Xia J., Zhou X., Xu S., Huang H., Zhang L., Zhou X., Du C. (2020). Risk Factors Associated With Acute Respiratory Distress Syndrome and Death in Patients With Coronavirus Disease 2019 Pneumonia in Wuhan, China. JAMA Intern. Med..

[2] Mækelæ† M.J., Reggev† N., Dutra N., Tamayo R.M., Silva-Sobrinho R.A., Klevjer K., Pfuhl G. (2020). Perceived efficacy of COVID-19 restrictions, reactions and their impact on mental health during the early phase of the outbreak in six countries. R. Soc. Open Sci..

[3] Kelly M.E., Duff H., Kelly S., Power J.E.M., Brennan S., Lawlor B.A., Loughrey D.G. (2017). The impact of social activities, social networks, social support and social relationships on the cognitive functioning of healthy older adults: A systematic review. Syst. Rev..

[4] Holt-Lunstad J. (2018). Why Social Relationships Are Important for Physical Health: A Systems Approach to Understanding and Modifying Risk and Protection. Annu. Rev. Psychol..

[5] Parra-Rizo M., Sanchís-Soler G. (2021). Physical Activity and the Improvement of Autonomy, Functional Ability, Subjective Health, and Social Relationships in Women over the Age of 60. Int. J. Environ. Res. Public Health.

[6] Cohn-Schwartz E., Vitman-Schorr A., Khalaila. R. (2021). Physical distancing is related to fewer electronic and in-person contacts and to increased loneliness during the COVID-19 pandemic among older Europeans. Qual. Life Res..

[7] Armitage R., Nellums L.B. (2020). COVID-19 and the consequences of isolating the elderly. Lancet Public Health.

[8] Seifert A., Hassler B. (2020). Impact of the COVID-19 pandemic on loneliness among older adults in Austria. Front. Sociol..

[9] McGinty E.E., Presskreischer R., Han H., Barry C.L. (2020). Psychological Distress and Loneliness Reported by US Adults in 2018 and April 2020. JAMA.

[10] Rajkumar R.P. (2020). COVID-19 and mental health: A review of the existing literature. Asian J. Psychiatry.

[11] World Health Organization (2020). Coronavirus Disease (COVID-2019) Situation Report—51.

[12] Sohrabi C., Alsafi Z., O’Neill N., Khan M., Kerwan A., Al-Jabir A., Iosifidis C., Agha R. (2020). World Health Organization declares global emergency: A review of the 2019 novel coronavirus (COVID-19). Int. J. Surg..

[13] Cohen-Mansfield J., Muff A., Meschiany G., Lev-Ari S., Aviv Israel T. Adequacy of Web-Based Activities as a Substitute for In-Person Activities for Older Persons during the COVID-19 Pandemic: Survey Study. https://www.jmir.org/2021/2/e27687/.

[14] Zubatsky M., Berg-Weger M., Morley J. (2020). Using Telehealth Groups to Combat Loneliness in Older Adults through COVID-19. J. Am. Geriatr. Soc..

[15] Fang Y., Chau A.K.C., Wong A., Fung H.H., Woo J. (2018). Information and communicative technology use enhances psycho-logical well-being of older adults: The roles of age, social connectedness, and frailty status. Aging Ment. Health.

[16] Shapira N., Barak A., Gal I. (2007). Promoting older adults’ well-being through Internet training and use. Aging Ment. Health.

[17] Yeshua-Katz D., Shapira S., Aharonson-Daniel L., Clarfield A.M., Sarid O. (2021). Matching Digital Intervention Affordances with Tasks: The Case of a Zoom and WhatsApp Mental Health Intervention for Seniors during the COVID-19 Pandemic. Health Commun..

[18] Fokkema T., Knipscheer K. (2007). Escape loneliness by going digital: A quantitative and qualitative evaluation of a Dutch experiment in using ECT to overcome loneliness among older adults. Aging Ment. Health.

[19] Silva P., Matos A.D., Martinez-Pecino R. (2020). Can the internet reduce the loneliness of 50+ living alone?. Inf. Commun. Soc..

[20] Mahlo L., Windsor T. (2020). Feasibility, Acceptability, and Preliminary Efficacy of an App-Based Mindfulness-Meditation Program Among Older Adults|The Gerontologist|Oxford Academic. Gerontologist.

[21] Segal-Engelchin D., Huss E., Sarid O. (2021). The Use of Online CB-ART Interventions in the Context of COVID-19: Enhanc-ing Salutogenic Coping. Int. J. Environ. Res. Public Health.

[22] Mewton L., Sachdev P.S., Andrews G. (2013). A Naturalistic Study of the Acceptability and Effectiveness of Internet-Delivered Cognitive Behavioural Therapy for Psychiatric Disorders in Older Australians. PLoS ONE.

[23] Titov N., Fogliati V.J., Staples L.G., Gandy M., Johnston L., Wootton B., Nielssen O., Dear B.F. (2016). Treating anxiety and depression in older adults: Randomised controlled trial comparing guided V. self-guided internet-delivered cognitive–behavioural therapy. BJPsych Open.

[24] Tomasino K.N., Lattie E.G., Ho J., Palac H.L., Kaiser S.M., Mohr D.C. (2017). Harnessing Peer Support in an Online Intervention for Older Adults with Depression. Am. J. Geriatr. Psychiatry.

[25] Shiovitz-Ezra S., Ayalon L. (2009). Situational versus chronic loneliness as risk factors for all-cause mortality. Int. Psychogeriatr..

[26] Masi C.M., Chen H.-Y., Hawkley L.C., Cacioppo J.T. (2010). A Meta-Analysis of Interventions to Reduce Loneliness. Pers. Soc. Psychol. Rev..

[27] Dickens A.P., Richards S.H., Greaves C.J., Campbell J.L. (2011). Interventions targeting social isolation in older people: A systematic review. BMC Public Health.

[28] Antonucci T.C., Ajrouch K.J., A Manalel J. (2017). Social Relations and Technology: Continuity, Context, and Change. Innov. Aging.

[29] Poscia A., Stojanovic J., La Milia D.I., Duplaga M., Grysztar M., Moscato U., Onder G., Collamati A., Ricciardi W., Magnavita N. (2018). Interventions targeting loneliness and social isolation among the older people: An update systematic review. Exp. Gerontol..

[30] Khosravi P., Rezvani A., Wiewiora A. (2016). The impact of technology on older adults’ social isolation. Comput. Hum. Behav..

[31] Baker S., Warburton J., Waycott J., Batchelor F., Hoang T., Dow B., Ozanne E., Vetere F. (2018). Combatting social isolation and increasing social participation of older adults through the use of technology: A systematic review of existing evidence. Australas. J. Ageing.

[32] Cheung G., Peri K. (2021). Challenges to dementia care during COVID-19: Innovations in remote delivery of group Cognitive Stimulation Therapy. Aging Ment. Health.

[33] Dikaios E., Sekhon H., Allard A., Vacaflor B., Goodman A., Dwyer E., Lavin-Gonzalez P., Mahdanian A., Park H., Walsh C. (2020). Connecting During COVID-19: A Protocol of a Volunteer-Based Telehealth Program for Supporting Older Adults’ Health. Front. Psychiatry.

[34] Office E.E., Rodenstein M.S., Merchant T.S., Pendergrast T.R., Lindquist L.A. (2020). Reducing Social Isolation of Seniors during COVID-19 through Medical Student Telephone Contact. J. Am. Med. Dir. Assoc..

[35] Shapira S., Yeshua-Katz D., Cohn-Schwartz E., Aharonson-Daniel L., Sarid O., Clarfield A.M. (2021). A pilot randomized con-trolled trial of a group intervention via Zoom to relieve loneliness and depressive symptoms among older persons during the COVID-19 outbreak. Internet Interv..

[36] Shapira S., Yeshua-Katz D., Goren G., Aharonson-Daniel L., Clarfield A.M., Sarid O. (2021). Evaluation of a Short-Term Digital Group Intervention to Relieve Mental Distress and Promote Well-Being Among Community-Dwelling Older Individuals During the COVID-19 Outbreak: A Study Protocol. Front. Public Health.

[37] Daitch C. (2018). Cognitive Behavioral Therapy, Mindfulness, and Hypnosis as Treatment Methods for Generalized Anxiety Disorder. Am. J. Clin. Hypn..

[38] Vanhuffel H., Rey M., Lambert I., Da Fonseca D., Bat-Pitault F. (2017). Contribution of mindfulness meditation in cognitive behavioral therapy for insomnia. Encephale.

[39] Goren G., Schwartz D., Friger M., Banai H., Sergienko R., Regev S., Abu-Kaf H., Greenberg D., Nemirovsky A., Ilan K. (2021). Randomized Controlled Trial of Cognitive-Behavioral and Mindfulness-Based Stress Reduction on the Quality of Life of Patients with Crohn Disease. Inflamm. Bowel. Dis..

[40] Kroenke K., Spitzer R.L., Williams J.B.W. (2001). The PHQ-9: Validity of a brief depression severity measure. J. Gen. Intern. Med..

[41] Neria Y., Besser A., Kiper D., Westphal M. (2010). A longitudinal study of posttraumatic stress disorder, depression, and generalized anxiety disorder in Israeli civilians exposed to war trauma. J. Trauma. Stress.

[42] Hughes M.E., Waite L.J., Hawkley L.C., Cacioppo J.T. (2004). A Short Scale for Measuring Loneliness in Large Surveys Results From Two Population-Based Studies. Res. Aging.

[43] Palgi Y., Shrira A., Ring L., Bodner E., Avidor S., Bergman Y., Cohen-Fridel S., Keisari S., Hoffman Y. (2020). The loneliness pandemic: Loneliness and other concomitants of depression, anxiety and their comorbidity during the COVID-19 outbreak. J. Affect. Disord..

[44] Broadhead W., Gehlbach S., de Gruy F., Kaplan B. (1988). The Duke-UNC Functional Social Support Questionnaire: Measure-ment of Social Support in Family Medicine Patients on JSTOR. Med. Care.

[45] Machón M., Larrañaga I., Dorronsoro M., Vrotsou K., Vergara I. (2017). Health-related quality of life and associated factors in functionally independent older people. BMC Geriatr..

[46] Applebaum A.J., Stein E.M., Lord-Bessen J., Pessin H., Rosenfeld B., Breitbart W. (2014). Optimism, social support, and mental health outcomes in patients with advanced cancer. Psycho-Oncol..

[47] Käll A., Jägholm S., Hesser H., Andersson F., Mathaldi A., Norkvist B.T., Shafran R., Andersson G. (2020). Internet-Based Cognitive Behavior Therapy for Loneliness: A Pilot Randomized Controlled Trial. Behav Ther..

[48] Torous J., Lipschitz J., Ng M., Firth J. (2020). Dropout rates in clinical trials of smartphone apps for depressive symptoms: A systematic review and meta-analysis. J. Affect. Disord..

[49] Löwe B., Unützer J., Callahan C.M., Perkins A.J., Kroenke K. (2004). Monitoring Depression Treatment Outcomes With the Patient Health Questionnaire-9. Med Care.

[50] Müller A., Arikian A., De Zwaan M., Mitchell J. (2011). Cognitive-behavioural group therapy versus guided self-help for compulsive buying disorder: A preliminary study. Clin. Psychol. Psychother..

[51] Chen Y.R.R., Schulz P.J. (2016). The Effect of Information Communication Technology Interventions on Reducing Social Isolation in the Elderly: A Systematic Review. J. Med Internet Res..

[52] Pynnönen K., Törmäkangas T., Rantanen T., Tiikkainen P., Kallinen M. (2018). Effect of a social intervention of choice vs. control on depressive symptoms, melancholy, feeling of loneliness, and perceived togetherness in older Finnish people: A randomized controlled trial. Aging Ment Health.

[53] Huxhold O., Hees E., Webster N.J. (2020). Towards bridging the grey digital divide: Changes in internet access and its predictors from 2002 to 2014 in Germany. Eur. J. Ageing.

[54] Pywell J., Vijaykumar S., Dodd A., Coventry L. (2020). Barriers to older adults’ uptake of mobile-based mental health interventions. Digit Health.

[55] Cohn-Schwartz E., Ayalon L. (2021). COVID-19 Protective Behaviors: The Role of Living Arrangements and Localities. J. Appl. Gerontol..

